# Inflammation of the Dentine

**Published:** 1856-04

**Authors:** J. Hamill


### INFLAMMATION OF THE DENTINE

BY J. HAMILL.

GENTLEMEN :—

Our subject is inflammation of dentine. We do not expect to present anything new upon this subject, because we have not lived long enough in the profession to enable us to hatch out any new theory, beside, as all theories should be in order to make them practicable, upon experience ; but we will present what we consider to be facts, in our own language, as near as perspicuity will permit, without either acknowledging authors or pretending to anything like priority of idea, or as one of our members jocularly remarked at a former meeting of this society, "we will scare up the game, and you must catch it," and I will add, or run it to its hole. For I tell you, gentlemen, there is game about this region of dentistry which has not yet been scared from its hiding place.

Many writers have noticed this subject but it has always been in connection with and collateral to some more immediate subject of discussion. We are not aware that it has ever been made the subject of especial thesis.

It is therefore with no little hesitation that we enter upon the discussion of it.

In the outstart of our remarks, we will refer yon briefly to a consideration of the anatomy and physiology of dentine ; and then take it up pathologically. Dentine, both in structure and position occupies a medium between crusta petrosa and enamel. It predominates largely over the former in mineral matter, but has much less of it than the latter.

These three structures crusta petrosa, dentine and enamel, are each produced from separate pulps, that of dentine being the first to make its appearance. It springs forth from the mucous membrane of the alveolar ridge in the form of a papilla, and is made of nucleated cells. These cells by the growth of thin nuclei, produce others, similar to themselves, both in structure and purpose.

"The papilla is soon contained in a follicle, and surrounded by a transparent granular blastema."

At the base of the follicle blood vessels and nerves are formed, which are furnished to each of the nuclei. These blood vessels are not necessarily concerned in the further development of these cells, seeing they possess that power inherently; but it is supposed, that, "by transudation through their coats, they furnish the fluid with which they are surrounded. This follicle eventually becomes a sack and then its contained papilla becomes really the pulp, and assumes the shape of the future tooth. The cells, during the growth of the pulp, have no regular arrangement, but when the process of calcification is about to commence they then arrange themselves in rows, beginning at the point of the pulp, and thence to every part of its circumference. These rows are not in straight lines; but pursue a tortuous course representing the greater and smaller curvatures of the tubuli of dentine. The curvatures are no doubt caused by the force with which the ends of the cells are butted together, and this too is undoubtedly the means by which absorption is induced at the ends of the cells, forming them into tubes. As the time approaches for the calcification of the tooth the blastema which surrounds the cells becomes changed to a granular mass.

The cells divide and subdivide, and by becoming elongated unite at their ends, thus forming tubular tissue. Each of these subdivisions of the cells contains one or more nuclei. These also become elongated and united at their ends, forming smaller tubes. The tubes formed by the union of the cells, become calcified, but those formed by the union of the nuclei remain open and are the tubuli of perfectly formed dentine.

Calcification, as the above described arrangement of the cells indicates, begins on the surface of the pulp, and proceeds inwards. As soon as the cells on the surface become hardened, those next within arrange themselves in rows, become enlarged, and by the reception of phosphate of lime are consolidated. As these changes take place, two or more of the more external cells may unite with one which is more internal, and thus form the branching appearance of the tubuli of dentine.

In this way calcification goes on till the whole mass of dentine is formed, leaving but a small portion of the pulp remaining in connection with the blood vessels and nerves.

Here then, gentlemen, we have dentine fresh from the forming hand of nature. One of the most wonderful and beautiful links in the chain of man's formation. Strong to bite upon yet as delicate as the lilly.

We do not know of any part throughout the whole range of human anatomy, which presents more beauties to the eye of the naturalist, when assisted by the microscope, than this structure. But is this beautiful structure we have traced through ist formation fully competent to withstand all the vicisitudes to which it may be subjected. Alas! we cannot with confidence affirm it can. Hereditary poison may have stung the very first cell that was produced for the development of the body of its possessor; and if so, the trail of the serpent will sooner or later be seen even here. This being the case, it becomes the philanthropist's duty to study the windings of this trail, and if possible avert the destruction which accompanies it.

This then brings us to a consideration of the pathological condition of dentine; or in other words, inflammation. Inflammation is the effect of irritation, irritation is produced by excitants.

The proximate causes then of inflammation may be divided into three general classes, viz: Vital or predisposing, mechanical and chemical. Vital or predisposing causes are an inherent principle of the dentine; and are the offspring of a constitution hereditarily bad, or of actual disease in the general system at the time of the formation of the teeth. Hence their development is entirely dependent upon the other three classes of causes above named.

Mechanical causes are also limited in their operation. If left to themselves they can only destroy the dentine by breaking it down and wearing it away; but if united with chemical causes there is no limit to the destruction which may be effected. We come then to chemical causes, the great and active principle of inflammation of dentine. Although they are greatly facilitated in their action by the co-operation of predisposing and mechanical causes; yet they possess the power and they wield it too with a vengence, of eating out and disorganizing the structure of dentine until its very life-springs are turned to corruption.

The source, whence these chemical causes spring has been the object of great solicitation. The secretions of the mouth have all been considered, when produced under certain conditions of the general system; and also local disturbing causes, as the receptacle of the solvent agents. That they are all more or less concerned in their production we cannot doubt; but which is most actively engaged in their production has not yet to our knowledge, been positively proven. Our own belief is that the mucus is more instrumental in their production than any of the other secretions. This seems to be the most natural conclusion, first from the fact that the mucus is known to be acid even in a normal state; and secondly from the fact that in almost all cases where a condition exists calculated to excite to greater supply of mucus than is natural or than can be neutralized by the saliva, we find inflammation. Another fact also in support of this is, that the teeth which occupy that part of the mouth which is not so frequently washed by the saliva, are generally the first to be attacked by inflammation. And where the enamel is dense enough to resist the ravages of these agents, we are sure to find it eating under the enamel at the necks of the teeth. In such cases you almost invariably find a deposite of claber-like mucus. This condition we are most apt to find in children; and the excess of mucus is attributable to the irritation consequent upon dentition.

In the adult, the proximate cause of inflammation must be the same as that in children, but the remote cause (dentition being completed) must be something else. And this we believe to be any thing which will produce irritation of the gums. "Their name is legion:" but we will only notice a few of such as are local, and, we think, most fruitful in the development of conditions calculated to produce inflammation.

Old roots of teeth will do it—badly constructed artificial teeth will do it—too much smoking will do it—and we are rather inclined to the belief that chewing tobacco will do it also. We do not wish to be understood as advancing the idea that tobacco will, by any chemical action in itself considered, produce irritation either in the gums or dentine, but we will show you presently that it can, as a mechanical agent, produce a condition favorable to the development of inflammation. Old roots of teeth, when they have lost their periosteal connection with the jaw, and are retained in the mouth by their connection with the gum only, are a fruitful cause of irritation. And owing to the sensitive condition of the gums, nothing is suffered by the person in whose mouth they are, to come in contact with them. This, then, taken in connection with their rough surfaces, enables them to retain all the mucus which is thrown out by the gums around them, in contact with the teeth that are next to them. They also, by the motions which they make every time the muscles of the mouth are brought into exercise, cause the gums to recede from the necks of the adjoining teeth. Thus we have a marked condition for inflammation, and one too which is met with very frequently in dental practice. Badly constructed artificial teeth not only excite the mucous glands to an unhealthy action, but retain that excess of mucus thrown out under the plate and clasps in contact with the teeth, and exclude the free circulation of saliva, which would otherwise wash it away, and neutralize its contained acid.

We said too much smoking was instrumental in producing a condition for inflammation, and so we have got ourself into the midst of the "*Tobacco question.*" But we don't care. We will give our opinion on the subject, and it is founded upon facts derived from personal experience. We have found it to be the case, that, when we smoked to excess, the mucous membrane of the mouth becomes very much irritated, and sometimes it extends even to the throat; but the roof of the mouth and along by the necks of the superior inscisor teeth generally suffers most. And we have found, too, that where this was persisted in for two or three days, if any part of these teeth, which had lost its enamel, were touched by an instrument, a decided sensation would be felt. This is our own experience with smoking; and although every other man's experience will, no doubt, differ from it, yet we think that if those who smoke would watch closely, they would occasionally find at least *inklings* of the same condition. We cannot speak so positively in reference to the chewing of tobacco, never having used it in that way; but observation has taught us, that, he who takes tobacco into the mouth and stows it away upon the labial surfaces of the molar teeth, thus excluding the saliva entirely from that part of the teeth, will soon find the gums and alveolar processes absorbed from their necks and roots. He will also find, that if an instrument be touched to that part of the teeth which is thus exposed, a thrill of pain will be sent throughout his whole system. If these wads of tobacco be taken from the mouth and examined, that part which lay in contact with the gums and teeth will be found coated with mucus. Here, then, we find the tobacco to be not only the exciting cause of the absorption of the gums and alveolar processes, but also the means by which the mucus is retained in contact with the dentine until inflammation is inevitable.

Chemical agents continue to operate upon the teeth as long as there are any in the mouth; but their effects are not felt so much in old persons as in the young and middle aged, from the fact that the teeth by this time have lost so much of their vitality. But where there is a healthy connection of the teeth with the jaw, if the crown of any of the teeth be broken or worn away so as to expose the dentine, we may find the same inflammatory condition. And sometimes it exists to such a marked degree that the teeth have to be removed; the frail state of the body not being able to endure its annoyance.

The nature of inflammation of dentine, like that of any other part of the animal structure, is to break down and disorganize the part in which it exists. It is generally confined to those points of the teeth which are best calculated to retain the solvent agent which produces it; but sometimes it pervades the whole body of the teeth. Decay of the teeth is not necessarily consequent upon its existence, but if it be permitted to progress unmolested it will necessarily terminate in decay. It may terminate in resolution and the part be restored to health again. This may be effected either by a change of condition in the general system or by the use of medicinal remedies applied locally. The nature of inflammation differs greatly in different kinds of teeth. It progresses most vigorously in that class of teeth which have a large proportion of animal tissue entering into their composition. It makes but little progress in teeth of the most dense structure.

The most striking peculiarity of this disease is the intense sensibility attending the first stage of its development.

In our review of the formation of dentine, we saw that the tubuli terminated in loops at its surface. These tubuli are the channels through which the nerves are transmitted, and, consequently, where these loops are, we must have a concentration of nerve fiber. This, then, will explain the reason why inflammation is attended by so much sensibility in its first stage. After a considerable portion of the crown of a tooth has been destroyed, we do not find so much sensibility except around the margin of the cavity, where the dentine and enamel unite, and at this stage of the disease it is greatest at the point where the two surfaces of the dentine come together, forming the cutting edge. And here we have a double concentration of nerve fiber.

We might multiply words upon this subject. We might have said less with more credit to ourself.

